# Effect comparison of neuroendoscopy versus microsurgery in the treatment of lateral ventricular tumors

**DOI:** 10.3389/fonc.2023.1008291

**Published:** 2023-07-24

**Authors:** Kai Li, Jianfeng Liang, Hongchuan Niu, Shuang Lan, Xiaoning Liang, Yuanli Zhao, Peng Zhao

**Affiliations:** ^1^ Department of Neurosurgery, Peking University International Hospital, Beijing, China; ^2^ Department of Operating Theatre, Peking University International Hospital, Beijing, China; ^3^ Department of Neurosurgery, PKUCare Zibo Hospital, Zibo, Shandong, China; ^4^ Department of Neurosurgery, Beijing Tiantan Hospital, Capital Medical University, Beijing, China

**Keywords:** lateral ventricle tumor, Neuro-Endoport, microsurgery, surgery, neuroendoscopy

## Abstract

**Purpose:**

We sought to reveal the clinical characteristics of lateral ventricle tumors and to evaluate the superior surgical procedure available.

**Methods:**

There involved a total of of 49 adult patients harboring lateral ventricle tumors in neurosurgery department of our hospital from January 2016 to March 2022. The patients enrolled were retrospectively analyzed, so are their clinical manifestations, pathological characteristics and surgical strategies. The patients were allocated into neuroendoscope group (11 cases) and microsurgery group (38 cases) according to the operation method. The two groups underwent a detailed evaluation of operation effectiveness and safety profile (operation time, intraoperative bleeding, surgical resection rate, postoperative complications) and economic indicators (postoperative hospital stay, hospital costs).

**Results:**

The neuroendoscope group demonstrated a markedly shorter operation time than the microsurgery group (p<0.05), with the amount of bleeding significantly less than the microsurgery group (p<0.01). However, there was no significant difference in the resection rate and postoperative complications between the two groups (p>0.05). Significant difference was found in the economic indicators (postoperative hospital stay and hospital costs) of the patients in the neuroendoscope group (p<0.05).

**Conclusion:**

Surgery intervention is regarded as the core treatment option for lateral ventricle tumors. Both microsurgery and neuroendoscopy are effective with safety profile. In the selected lateral ventricle tumor surgery, the application of neuroendoscopic surgery showed promising results, in terms of less intraoperative bleeding, and shorter operation time, postoperative hospital stays, and hospital costs. The selection of surgical approach and methods for lateral ventricle tumors is principally depended on the experience of neurosurgeon concerning the surgical approach and related neuroanatomy.

## 1 Introduction

Lateral ventricle tumor refers to ventricular tumor that develops in the lateral ventricle, accounting for about 0.8-1.6% of intracranial tumors (1, 2). The disease can be originally divided into primary and secondary lateral ventricle tumor. The former originates from the ventricular wall and intraventricular tissue, and the latter from adjacent brain tissue and invades the lateral ventricle. The ever-present challenge of patients with lateral ventricular tumors is that no special clinical symptoms showed in the early stage since the tumors grow in the ventricular cavity. Following the enlarging process, the tumor blocks the cerebrospinal fluid circulation pathway and invades the adjacent structures, the corresponding clinical symptoms appear (3, 4). Surgical resection is currently identified as the preferred method for patients with lateral ventricle tumors. However, Key nerves and blood vessels near the deep structure and rich blood supply to increase the risk of surgery. For most neurosurgeons, lateral ventricle tumor resection is still a challenging operation. Despite that both microsurgery and endoscopic surgery are available, how to select the appropriate surgical approaches is still an ongoing debate, such as transcortical approach and transcallosal approach. There are few comparative studies on the surgical methods of microscopic surgery or endoscopic surgery for lateral ventricle tumors in the past. From January 2016 to March 2022, we operated on 49 adult patients with lateral ventricle tumors and retrospectively analyzed their clinical manifestation, surgical plan, surgical effect, safety and economic indicators, in order to offer theoretical insight for the selection of surgical methods in clinical practice.

## 2 Materials and methods

### 2.1 General data

**Figure 1:**
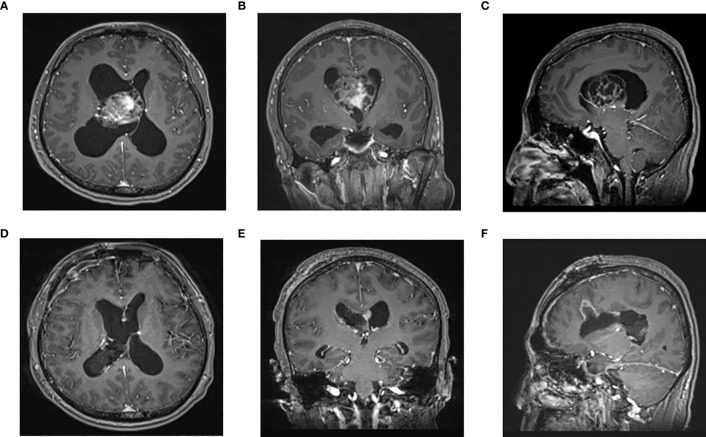
Enhanced MRI of head in one patient in endoscopic group before operation **(A–C)** and 3 days after operation **(D–F)**: The tumor was located in the right lateral ventricle. The tumor was resected satisfactorily and the brain channel recovered well after operation. **(A)** Preoperative axial **(B)** Preoperative coronal **(C)** Preoperative sagittal **(D)** Postoperative axial **(F)** Postoperative coronal F Postoperative sagittal.

**Figure 2:**
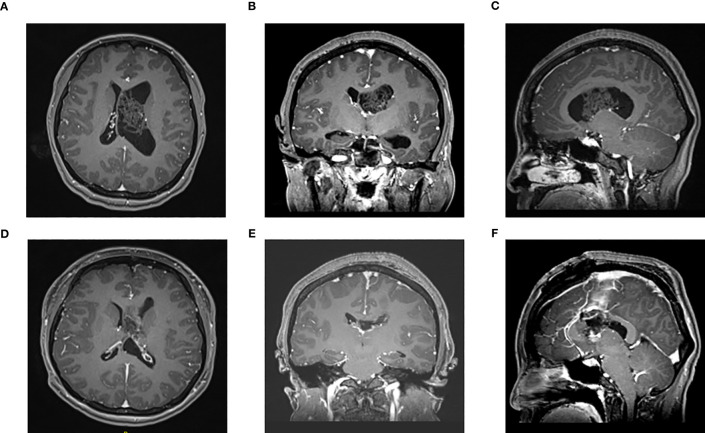
Enhanced MRI of head in one patient in the microscope group before surgery **(A–C)** and 3 days after surgery **(D–F)**: The tumor was located in the left lateral ventricle. **(A)** Preoperative axial **(B)** Preoperative coronal **(C)** Preoperative sagittal **(D)** Postoperative axial **(E)** Postoperative coronal **(F)** Postoperative sagittal.

**Figure 3:**
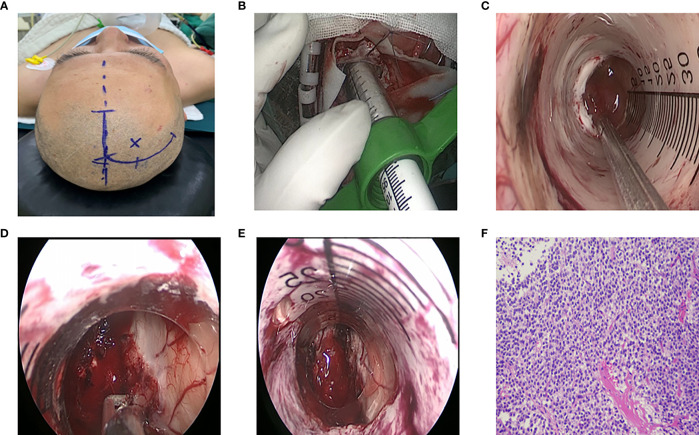
Surgical procedure of endoscopic treatment for intraventricular tumors. **(A)** Right frontal surgical incision. **(B)** Endoport retractor. **(C)** Observe the tumor boundary under endoscope. **(D)** The blood supply of tumor was coagulated by bipolar electrocoagulation, and the tumor was excised by suction. **(E)** Tumor resection, adequate hemostasis, **(F)** Postoperative pathology: central neurocytoma, WHOII grade.

**Figure 4:**
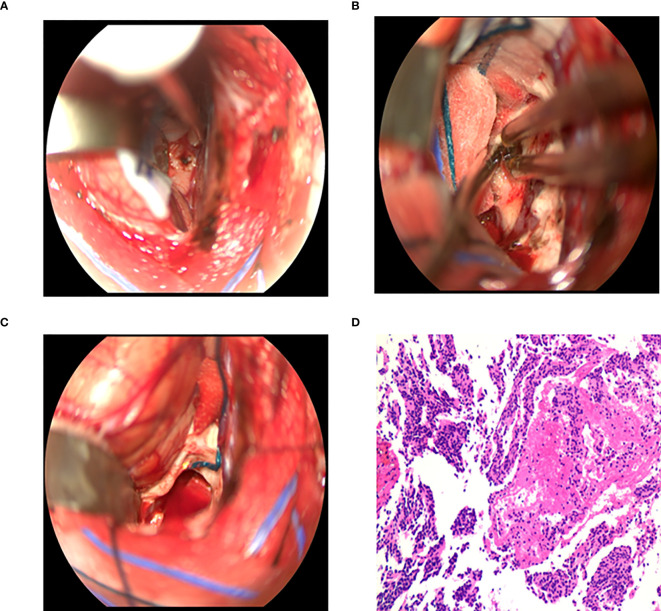
Surgical procedure of intraventricular tumors in the microscopic group. **(A)** The corpus callosum was incised 1.5 - 2 cm to expose the tumor. **(B)** The blood supply of tumor was coagulated by bipolar electrocoagulation, and the tumor was excised by suction. **(C)** Tumor resection and full hemostasis. **(D)** Postoperative pathology: central neurocytoma, WHOII grade.

Clinical data of 49 eligible adult patients harboring lateral ventricle tumors in the neurosurgery department of our hospital from January 2016 to March 2022 were retrospectively analyzed. Inclusion criteria (1): tumor located completely or mainly in ventricle (2); The surgery was the first surgery without radiotherapy and/or chemotherapy (3); Complete clinical data. If the diameter of ventricular tumor is more than 5.5cm, microsurgery is selected; if the diameter is less than 5.5cm, microsurgery or neuroendoscopic surgery is selected according to the location of the tumor and the experience of the operator. The patients were allocated into neuroendoscope group (11 cases) and microsurgery group (38 cases) according to the operation method. All 49 patients signed informed consent, and this study complied with the principles of the Declaration of Helsinki. The clinical background of the patients was collected, including age, gender, initial symptoms, pathogenic site (**Figures 1A–F**, **2A–F**), pathological type (**Figures 3F**, **4D**), operation effectiveness and safety indicators (operation time, intraoperative bleeding, surgical resection rate, postoperative complications), and economic indicators (postoperative hospital stay, hospital costs).

### 2.2 Surgical methods

#### 2.2.1 Microsurgery procedure

Behind the frontal or temporal or parietal occipital bone flap, the cerebral cortex was cut 3 to 4cm or the cortex was fistulated into the lateral ventricle, and the brain tissue was pulled away to remove the tumor. Through the anterior approach of the corpus callosum, a “U” shaped incision near the midline was performed to form a 6cm×4cm bone flap. The posterior edge of the bone flap was located in the coronal suture. And the dura mater was turned to the side of the sagittal sinus. The small reflux vein between the brain surface and the sagittal sinus was cut off by electrocoagulation, and the cut side was close to the brain surface. The right lobe of the brain was stretched to expose the corpus callosum along the longitudinal fissure, and surgeons needed to protect the pericallosal artery during exposure to avoid damage. The corpus callosum was cut about 2cm into the lateral ventricle (**Figure 4A**) and the tumor was removed (**Figures 4B, C**). During the operation, the interventricular foramen was protected to prevent blood from flowing into the contralateral ventricle or the third ventricle. At the same time, the stria colliculus vein and internal cerebral vein were strictly protected, followed by electro coagulating the choroid plexus, to reduce the secretion of cerebrospinal fluid after the operation.

#### 2.2.2 Procedures under neuroendoscope

With the help of neuronavigation system, the approach to enter was determined. A curved incision (**Figure 3A**) was made around the endoscopic insertion point to free the small bone flap. The bone flap was about 3cmx3cm in size. And the dura was cut in an arc. After electrocoagulation in the avascular area on the cortical surface, the lateral ventricle was punctured with a brain needle. After successful puncture, the brain needle was withdrawn, and the ventriculoscope sheath or Endoport channel (**Figure 3B**) along the direction of the brain needle was placed. The tumor was removed according to the blood supply, size and origin of the tumor. The neuroendoscope was withdrawn after there had been no active bleeding, and the guider was withdrawn slowly along the original channel (**Figures 3C-E**). Moreover, the dura was sutured layer by layer after there had been no bleeding, and the bone flap was reset.

### 2.3 Statistical analysis

SPSS 19.0 software was applied for statistical analysis. Continuous variables were tested for normal distribution using Shapiro Wilk method. Data with normal distribution was described by mean ± standard deviation (x ± s). The difference in means between the two sample was compared using independent sample t-test. Categorical variables were described by the number of cases and constituent ratio, and the differences between groups were tested by x^2^ test. A p<0.05 was defined as statistically significant (two tails).

## 3 Results

### 3.1 Preoperative characteristics

Demographic data and clinical symptoms are shown in **Table 1**. Among the 49 adult patients with lateral ventricle tumors, 21 were males and 28 were females, aged from 20 to 75 years, with an average of 42.1 years. The diameter of the tumor was 1cm-8.3cm, with an average of 4.3cm. The most common symptoms included headache/dizziness in 30 cases (61.2%), nausea/vomiting in 10 cases (20.4%), unintentional discovery of the disease in 7 cases (14.3%), visual impairment in 6 cases (12.2%), limb numbness in 5 cases (10.2%), limb weakness in 2 cases (4.1%), memory impairment in 2 cases (4.1%), gait instability in 2 cases (4.1%), seizures in 1 case (2.0%), disturbance of consciousness in 1 case (2.0%) and urinary incontinence in 1 case (2.0%). The course of disease ranged from 3 days to 3 years, with an average of 4 months.

**Table 1:** Characteristics of 49 patients with lateral ventricle tumors.

Characteristics	N (%)
Demographics	
Mean age, (years) (range)	42.1 (20–75)
Sex (male/female)	21/28
Tumor features	
Mean size, (cm) (range)	4.3 (1.0-8.3)
Tumor diameter in neuroendoscope group 0-3(cm)	4 (8.2%)
Diameter 3-5(cm)	5 (10.2%)
Diameter >5(cm)	2 (4.1%)
Tumor diameter in microsurgery group 0-3(cm)	6 (12.2%)
Diameter 3-5(cm)	18 (36.7%)
Diameter >5(cm)	14 (28.6%)
Location	
Left	27 (55.1%)
Right	21 (42.9)
Both sides	1 (2.0%)
The frontal horn	6( 12.2%)
The body	20 (40.8%)
The atrium	20 (40.8%)
The body, the atrium	2 (4.1%)
The body, the atrium, the temporal horn	1 (2.0%)
Symptoms	
Headache and dizziness	30 (61.2%)
Nausea and vomiting	10 (20.4%)
Unintentional discovery of the disease	7 (14.3%)
Visual impairment	6 (12.2%)
Limb numbness	5 (10.2%)
Limb weakness	2 (4.1%)
Memory impairment	2 (4.1%)
Gait instability	2 (4.1%)
Seizures	1 (2.0%)
Urinary incontinence	1 (2.0%)
Disturbance of consciousness	1 (2.0%)
Mean symptoms duration, (months)	4.0
Range	3 days-3years

### 3.2 Surgical features and pathology

There were 39 cases in the microsurgery group, with 15 cases undergoing frontal transcortical approach, 4 undergoing anterior transcallosal approach, and 3 undergoing temporal transcortical approach. Transcortical approach of parietal occipital lobe was performed in 16 cases. On the other hand, there were 11 patients in the neuroendoscope group, 3 of whom were treated using ventriculoscopy and 8 with Endoport-assisted neuroendoscopy. The operative approaches were frontal cortex approach in 8 cases and parietal occipital triangle approach in 3 cases. Postoperative pathology: meningioma in 17 cases (34.7%), central neurocytoma in 13 cases (26.5%), glioma in 8 cases (16.3%) (low grade: 5 cases, high grade: 3 cases), subependymal tumor in 4 cases (8.2%), and ependymoma, epidermoid cyst, low-grade malignant mesenchymal tumor, germinoma, choroid plexus granuloma, metastasis (lung cancer metastasis) and neuroepithelial cyst in 1 case each (2.0%). Surgical approach and pathology are summarized in **Table 2**.

**Table 2:** Surgical approach and pathology.

Surgical approach and pathology	N (%)
Neuroendoscopy Transfrontal approach	8 (16.3%)
Transparietal occipital triangle approach	3 (6.1%)
Microsurgery Frontal transcortical approach	15 (30.6%)
Anterior transcallosal approach	4( 8.2%)
Transcortical approach of temporal lobe	3 (6.1%)
Transcortical approach of parietooccipital lobe	16 (32.7%)
Pathology	
Meningioma	17 (34.7%)
Central neurocytoma	13 (26.5%)
Low-grade glioma	5 (10.2%)
Subependymal tumor	4 (8.2%)
High-grade glioma	3 (6.1%)
Epidermoid cyst	1 (2.0%)
Low-grade malignant mesenchymal tumor	1 (2.0%)
Germ cell tumor	1 (2.0%)
Choroid plexus granuloma	1 (2.0%)
Ependymoma	1 (2.0%)
Metastatic tumor (brain metastasis of lung cancer)	1 (2.0%)
Neuroepithelial cyst	1 (2.0%)

### 3.3 Comparison of two surgical methods

**Table 3** laid out the comparison of general data between the two groups. There was no significant difference in the age and gender of patients in the two groups (P=0.533, P=0.379). Based on the comparison of economic indicators (**Table 3**), the length of hospital stay (P=0.039) and hospitalization cost (P=0.021) of patients in the neuroendoscopy group were lower than those in the microsurgery group. Results of comparison of efficacy and safety showed that the operation time in the neuroendoscope group was significantly shorter than that in the microsurgery group (P=0.021<0.05). The intraoperative bleeding in the neuroendoscope group was significantly less than that in the microsurgery group (P=0.001<0.01). However, there was no significant difference in resection rate (P=0.820) and postoperative complications (P=0.178) between the two groups (p>0.05). In the neuroendoscope group, 1 case had bleeding in the puncture tract and 1 case had intracranial infection. In the microsurgery group, there were 16 cases of complications, 3 of epilepsy, 3 of epidural hematoma, 2 of intracranial infection, 2 of isolated temporal angle, 2 of death, 1 of cerebral infarction, 1 of intracranial hematoma, 1 of diabetes insipidus and 1 of brain swelling.

**Table 3:** Comparison of clinical data between neuroendoscopy group and microsurgery group.

Item	Neuroendoscopy group (11 cases)	Microsurgery group (38 cases)	P value
Age (year, x±)	45.2 ± 16.1	41.2 ± 12.3	0.533
Male/female(cases)	6/5	15/23	0.379
Postoperative hospital stay (day)	11.2 ± 4.4	16.8 ± 10.6	0.039
Hospitalization cost (RMB,ten thousands)	7.5 ± 3.3	10.4 ± 4.2	0.021
Operation time (min)	255.4 ± 168.5	315.9 ± 93.5	0.021
Intraoperative blood loss (ml)	96.3 ± 73.5	459.5 ± 455.4	0.001
Surgical resection rate (%)	99.6 ± 1.51	98.5 ± 5.2	0.820
Postoperative complications (case)	2	16	0.136

Two groups of patients according to tumor size, to compare the operation time, intraoperative blood loss and postoperative complications When the tumor diameter was less than 5 cm, the endoscopic group had less operation time and intraoperative bleeding than the tumor group, but the postoperative complications were not significantly reduced (**Table 4**)

**Table 4:** Comparison of surgical effects of tumors with different diameters.

Item	Diameter (cm)	Neuroendoscopy group	Microsurgery group	P value
Operation time (min)	0<D<5 D≥5	221.8 ± 62.8 406.5 ± 443.4	305.8 ± 90.6 332.6 ± 99.5	0.016 0.531
Intraoperative blood loss (ml)	0<D<5 D≥5	84.4 ± 77.2 110.0 ± 56.6	459.5 ± 455.4 675.0 ± 671.0	0.001 0.011
Postoperative complications (case)	0<D<5 D≥5	1 1	10 6	0.097 0.849

## 4 Discussion

### 4.1 Clinical presentation

The ever-present challenge of patients with lateral ventricular tumors is that no special clinical symptoms showed in the early stage since the tumors grow in the ventricular cavity. Following the enlarging process, the tumor blocks the cerebrospinal fluid circulation pathway and invades the adjacent structures, the corresponding clinical symptoms appear (3, 4). Li-Feng Chen et al. (5) reported that preoperative symptom duration ranged from 2 days to 2 years (mean, 5.5 months). The most common signs and symptoms are associated with increased intracranial pressure (ICP), including headache, nausea, vomiting, and sleepiness. Gokalp et al. (2) depicted that 42.9% of patients had papilledema, 35.7% had headache, 25% had dyskinesia, 25% had sensory disturbance, and 22.3% had nausea/vomiting. Study supported by Sherif M. Elwatidy et al. (3) showed that there included headache (29 cases, 69%), nausea/vomiting (16 cases, 38%), visual impairment (10 cases, 24%), and seizes (7 cases, 17%) in patients with lateral ventricular tumors. In our study, the average duration of preoperative symptoms was 4.0 months, and the longest was 3 years. The most common symptoms were related to increased ICP and hydrocephalus after cerebrospinal fluid circulation obstruction, including headache/dizziness in 30 cases (61.2%), nausea/vomiting in 10 cases (20.4%), visual impairment in 6 cases (12.2%). And 7 cases (14.3%) were asymptomatic in the early stage and found unintentionally during physical examination. The minimum and maximum diameter of the tumor was 1cm and 8.3cm, resulting in the difficulty to early identify the tumor.

### 4.2 Pathology

Lateral ventricle tumors account for about 0.8-1.6% of intracranial tumors (1, 2). And benign tumors account for 64%, moderate malignant tumors 15%, and malignant tumors 21% of intraventricular tumors (2). The nature of lateral ventricular tumors is more uncertain and likely to vary by the age of onset. For instance, choroid plexus papilloma and malignant small cyanocytoma are common in children. In contrast, pilocytic astrocytoma, subependymal giant cell astrocytoma and diffuse low-grade astrocytoma mostly occur in patients aged 6-30 years. Meningiomas, metastases, and high-grade gliomas are commonly seen in patients over the age of 30. According to the study of Gokalp et al. (2), the most common pathologies were ependymoma (25%), astrocytoma (21.4%), and oligodendrocytoma (7.1%). In another study of Marvin Darkwah et al. (6), Malignancies (metastasis or WHO grade III/IV tumor) were uncommon and only diagnosed in 7 (13.5%). And in the present study, the most common tumors were 17 meningiomas (34.7%), 13 central neurocytomas (26.5%), 8 gliomas (16.3%) (5 low-grade, 3 high-grade), and 4 subependymal tumors (8.2%). Metastatic tumor (brain metastasis of lung cancer) occurred in a 75- year-old male.

### 4.3 Surgical methods and approaches

#### 4.3.1 Comparison between microsurgery and neuroendoscope

Surgery for intraventricular tumors remains a controversial and evolving field (7). At present, the surgical treatment of lateral ventricle tumors depends on bone flap craniotomy under microsurgery. Open surgery continues to be the gold standard, especially in large and highly vascularized lesions in which endoscopy still has a limited role (7). Althrough craniotomy is acknowledged as the golden standard, especially in the lesions with large tumor diameter and abundant blood vessels, the role of neuroendoscopy is largely limited. Under the microsurgery, the operation not only has clear visual field exposure, complete hemostasis, high total tumor resection rate, and less complications, but can clearly identify the anatomical structure, and protect the thalamus, striated veins, basal nuclei and other important structures. However, there are several disadvantages of microsurgery. A larger stoma is usually required to better expose the tumor. The traditional brain pressure plate is often utilized during the operation. Due to uneven stress and insufficient protection of the operation channel, it is easy to cause severe brain edema after the operation, which may cause great damage to the brain tissue, and the probability of epilepsy and neurological loss after the operation may increase (8). Moreover, due to the tubular visual field of the microsurgery and the deep location of the ventricular tumor, the exposure of the operating field is limited, which affects the total resection of the tumor (9).

In recent years, as the advances of neuroendoscope instruments and neuroendoscope technology, the application of neuroendoscope in ventricular lesions has expanded from biopsy of ventricular tumors to complete resection of ventricular tumors by neuroendoscope. Advantages of neuroendoscope include (9–11): 1) compared with the microsurgery, neuroendoscope provides a visual field that can be viewed directly and from multiple angles, with good illumination. It can effectively help identify the details of the tumor and the surrounding important tissues, improve the total tumor resection rate, and help avoid nerve function damage. 2) During neuroendoscopic surgery, the surgical channel is relatively fixed, and the pulling force on the brain tissue is small. And the movement of surgical instruments is effectively limited to cause damage to the surrounding tissues, which reduces the probability of severe brain edema and epilepsy after surgery. 3) The cerebrospinal fluid circulation channel can be reconstructed while the tumor is removed. There are several disadvantages of neuroendoscope (12). Firstly, neurosurgeons need to receive specific technical training. The operation of instruments is not as convenient and skilled as that under the microsurgery, and it is difficult to deal with intraoperative bleeding (13). Secondly, it is difficult to treat tumors with large diameter or abundant blood vessels in the lateral ventricle (7). Some scholars believe that the indications for the application of neuroendoscopy are that the maximum diameter of the tumor is <2.5cm, with clear boundary, less adhesion, narrow pedicle and free in the ventricle (14). However, the maximum diameter of the tumor in this group of cases was 5.2cm. Neuro-Endoport channel was used during neuroendoscopy, and the surgeon can perform with both hands to complete the operation. On the basis of multi-angle movement of the channel, greater exposure space could be obtained the tumor could be resected in blocks (15–17).

Our experience based on the study is the following: 1) in order to avoid brain tissue injury and important tissue contusion around the interventricular foramen, intraoperative neuronavigation and intraoperative ultrasound can be applied together to more accurately plan the surgical path, and the use of ultrasonic attractor and needle electrode can improve the surgical efficiency (15). 2) Neuro-Endoport technology provides an independent surgical channel, which is able to obtain a larger exposure space. Furthermore, endoscopes, aspirators and conventional surgical instruments are placed, hence the operator can perform with both hands. 3) The operation should be done with caution to protect the important structures around the ventricles and deep veins, especially the internal cerebral veins and the veins of the colliculus. 4) The shape, size, blood supply, pedicle and adjacent structures of the tumor should be closely observed after the neuroendoscope enters the ventricle, to firstly deal with the base and blood supply vessels of the tumor. If the tumor originates from the midline, the tumor could be removed after identifying the midline structure (such as septum pellucidum and the inner side of the interventricular foramen) to disconnect the tumor base. If it originates from the periphery of the ventricular wall, the surrounding tissues should be closely protected before tumor resection. If the tumor has abundant blood supply, identify the normal anatomical structure, electrocoagulate the blood supply vessels first, and then remove the tumor as a whole. 5) The application of endoscope support arm is conducive to simultaneous operation of both hands and the coordination of electrocoagulation, shearing, pulling and other operations. 6) During the operation, the cerebrospinal fluid passage can be closed with cotton pieces, and the bleeding enters the cerebrospinal fluid circulation. 7) When the tumor diameter was less than 5 cm, the endoscopic group had less operation time and intraoperative bleeding than the tumor group, but the postoperative complications were not significantly reduced. Our clinical experience is also that endoscopic surgery is more suitable for tumors <5 cm in diameter. If the diameter of the tumor is too large, improper pushing and pulling of the tumor will not only damage the ventricular wall and thalamostriate vein, but also cause postoperative coma. Once the blood supply vessels of the tumor are ruptured, intraventricular hemorrhage may occur before the tumor is completely removed, and the consequences are quite serious.

In this study, the economic indicators, including the postoperative hospital stay and hospital costs of patients in the neuroendoscopy group were lower than those in the microsurgery group. As for the efficacy and safety profile, the operation time of neuroendoscope group was shorter than that of microsurgery group, and the amount of intraoperative bleeding was significantly less than that of microsurgery group. The microsurgery group had 16 cases of complications, including 2 cases of death. Among the dead cases, one case received the anterior approach of the corpus callosum. After the operation, the patient suffered from coma and severe diencephaledema, and finally died. It was considered to be caused by the injury of the internal cerebral vein and the great cerebral vein. Another dead case was treated via the parieto occipital cortical approach. After the operation, the patient continued to have grand mal seizures, and finally died of severe cerebral ischemia and swelling. There was no difference in resection rate and postoperative complications between the two groups. There were no postoperative deaths in the neuroendoscope group (0/11), while 2 postoperative deaths occurred in the microsurgery group (2/38). Microsurgery and neuroendoscope are both optional methods for lateral ventricle tumor surgery, mainly based on the experience of neurosurgeon in the surgical approach and related neuroanatomy. Further technical improvement is needed to improve the application of neuroendoscopy in lateral ventricle tumor surgery (7, 18).

#### 4.3.2 Surgical approaches

Surgical approaches for lateral ventricle tumors are mainly divided into transcortical or transcallosal approach, and the selection of appropriate approach has been the topic of debate (6). There are currently four common surgical approaches (6, 19).

##### 4.3.2.1 Parieto occipital transverse approach

This approach is preferred for tumors situated entirely within the atrium, posterior part of the body of the lateral ventricle, Atrial tumors extending into the occipital horn can also be approached using this route. The parieto occipital or interparietal sulcus approach can be used to avoid language dysfunction in the dominant hemisphere. The longitudinal cortex fistula in the parietal lobe 4 to 5cm posterior to the central sulcus to the parieto occipital sulcus is performed. The operation is performed behind the sensory area and above the angular gyrus and supramarginal gyrus to avoid damaging the angular gyrus and supramarginal gyrus. Gerstmann’s syndrome was reported in one third of patients undergoing this approach, when the dominant hemisphere is involved. Apraxia and acalculia m ay also occur (12).

##### 4.3.2.2 Frontal transcortical approach

This approach is suitable for lesions of the frontal horn and the anterior port ion of the body of the lateral ventricle. Frontal transcortical approach is easy to expose the anterior choroidal artery. During the transfrontal approach, the cortex is usually cut along the running direction of the middle frontal gyrus at 3cm in front of the central anterior gyrus to avoid damaging the language center of the inferior frontal gyrus of the dominant hemisphere (20).

##### 4.3.2.3 Temporal transcortical approach

Transcortical approach is used for tumors in the middle or posterior third of the temporal horn.Temporal transcortical approach is used for transverse cortical fistulation in the posterior 1/3 of the middle temporal gyrus. Parallel visual irradiation can reduce the incidence of postoperative hemianopia, facilitate the treatment of tumor blood supply, and minimize postoperative visual field defects (20).

##### 4.3.2.4 Corpus callosum approach

This approach is used for tumors located in the anterior horn and middle part of the body of the lateral ventricles. In the absence of ventriculomegaly, this approach is easier to perform and is superior to the anterior transcortical approach when the tumor is small, located near the midline, and does not require excessive hemispheric retraction. Compared with the transcortical approach, the transcallosal approach has the advantages of easy access to the lateral ventricle, no cortical incision, and reduced risk of postoperative seizures. Advantages of the corpus callosum approach include: 1) make full use of the natural anatomical space of the brain to approach and expose the tumor without damaging the cerebral hemisphere; 2) low incidence of postoperative epilepsy; 3) no important anatomical structure and little damage to physiological function; 4) longitudinal incision of the corpus callosum for 2cm do not greatly affect the information transmission of the bilateral hemispheres. However, the available length of the corpus callosum for incision is limited, about 2 to 2.5cm. It is difficult to reach the tumors in the triangle, posterior, occipital and temporal angle (15, 20).

Patients undergoing transcortical surgery are prone to have postoperative seizures (3, 20). However, some investigations have shown that seizures mainly occur in patients through the transcallosal approach. It is reported that the disadvantages of transcallosal approach include temporary silence and sacrifice of bridging vein in longitudinal fissure path, resulting in postoperative brain swelling and infarction (6, 21). Nevertheless, Sherif M. Elwatidy and Ulas Cikla believed that with the improvement of microscopy, the risk of epilepsy and other complications after transcortical approach and transcallosal approach may be reduced. There was no difference in postoperative cognitive function between the transcortical and transcallosal surgical approaches (22).

The first or second approach is available for neuroendoscope, and the abovementioned four approaches can be selected for microsurgery. Interhemispheric transcallosal and transcortical approaches were the best surgical access routes (20). However, whether microsurgery or endoscopic surgery, the surgical approach should be determined depending on a variety of other factors, including tumor location, cerebral ventricle and tumor size, location of arterial blood supply, pathological characteristics of the tumor, preoperative neurological deficit and experience of surgeon (19, 23).

## 5 Conclusion

Surgery intervention is regarded as the core treatment option for lateral ventricle tumors. Both microsurgery and neuroendoscopy are effective with safety profile. In the selected lateral ventricle tumor surgery, the application of neuroendoscopic surgery showed promising results, in terms of less intraoperative bleeding, and shorter operation time, postoperative hospital stay, and hospital costs. The selection of surgical approach and methods for lateral ventricle tumors is principally depended on the experience of neurosurgeon concerning the surgical approach and related neuroanatomy.

Neuroendoscopy can be a safe and effective tool for the resection of intraventricular tumors. Proper patient selection and a specially trained neurosurgeon are essential. Currently, the main limitation continues to be a larger size or vascularization of the lesion. In the future, endoscopic resection will become more popular.

## Data availability statement

The original contributions presented in the study are included in the article/supplementary material. Further inquiries can be directed to the corresponding author.

## Author contributions

KL and JL carried out the research. KL, JL and SL participated in data analysis. KL and HN drafted the manuscript. YZ and PZ critically reviewed the overall manuscript as well as supervised the study. All authors contributed to the article and approved the submitted version.

## References

[1] Lapras C Deruty R Bret PH. Tumours of the lateral ventricles. In: Symon L, editor. Advances and technical standards in neurosurgery, vol. 11. New York: Springer (1984). p. 103–67. 10.1007/978-3-7091-7015-1_5 6536266

[2] Gokalp HZ Yuceer N Arasil E Deda H Attar A Erdogan A. Tumors of the lateral ventricle: a retrospective review of 112 cases operated upon 1970–1997. Neurosurg Rev (1998) 21(2–3):126–37. doi: 10.1007/BF02389318 9795947

[3] Elwatidy Sherif M Albakr Abdulrahman A Al Towim Abdullah A Malik Safdar H. Tumors of the lateral and third ventricle: surgical management and outcome analysis in 42 cases. Neurosci (Riyadh) (2017) 22(4):274–81. doi: 10.17712/nsj.2017.4.20170149 PMC5946376 29057852

[4] Mei GH Liu XX Zhou P Shen M. Clinical and imaging features of subependymal giant cell astrocytoma: report of 20 cases. Chin Neurosurg Jl (2017) 3:14. doi: 10.1186/s41016-017-0077-4

[5] Chen L-F Yang Y Ma X-D Yu X-G Xu B-N Zhou D-B. Operative management of intraventricular central neurocytomas: an analysis of a surgical experience with 32 cases. Turk Neurosurg (2016) 26(1):21–8. doi: 10.5137/1019-5149.JTN.11356-14.2 26768865

[6] Darkwah Oppong M Müller O Jabbarli R Dammann P Sure U El Hindy N. Intraventricular mass lesions: still a question of surgical approach? J Clin Neurosci (2017) 43:157–62. doi: 10.1016/j.jocn.2017.05.036 28625588

[7] Ibáñez-Botella G Segura M De Miguel L Ros B Arráez MÁ. Purely neuroendoscopic resection of intraventricular tumors with an endoscopic ultrasonic aspirator. Neurosurgical Rev (2019) 42(4):973–82 doi: 10.1007/s10143-018-1011-8 30019320

[8] Kutlay M Durmaz MO Kırık A Yasar S Ezgu MC Kural C. Resection of intra- and paraventricular malignant brain tumors using fluorescein sodium-guided neuroendoscopic transtubular approach. Clin Neurol Neurosurg (2021) 207:106812. doi: 10.1016/j.clineuro.2021.106812 34280673

[9] Engh JA Lunsford LD Amin DV Ochalski PG Fernandez-Miranda J Prevedello DM. Stereotactically guided endoscopic port surgery for intraventricular tumor and colloid cyst resection. Neurosurgery (2013) 67(3 Suppl Operative):ons198–205. doi: 10.1227/01.NEU.0000382974.81828.F9 20679929

[10] Nanda A Bir SC Maiti T Konar S. Intraventricular meningioma: technical nuances in surgical management. World Neurosurg (2016) 88:526–37. doi: 10.1016/j.wneu.2015.10.071 26548837

[11] Akiyama Y Wanibuchi M Mikami T Horita Y Komatsu K Suzuki K. Rigid endoscopic resection of deep-seated or intraventricular brain tumors. Neurological Res (2015) 37(3):278–82. doi: 10.1179/1743132814Y.0000000439 25204627

[12] Mazher S Imran M Ashraf J Ahmed A Shah IU Zulfiqar F. Outcome of open transcortical approach in the management of intraventricular lesions. J Coll Physicians Surgeons–Pakistan (2013) 23(12):857–61. 24304988

[13] Mohanty A Thompson BJ Patterson J. Initial experience with endoscopic side cutting aspiration system in pure neuroendoscopic excision of large intraventricular tumors. World Neurosurg (2013) 80(5):655.e15–655.e6.55E21. doi: 10.1016/j.wneu.2012.11.070 23207734

[14] Elbabaa SK. Ventricular neuroendoscopic surgery: lessons learned from the literature. World Neurosurg (2016) 88:646–8. doi: 10.1016/j.wneu.2015.11.019 26608384

[15] Gaab MR Schroeder HW. Neuroendoscopic approach to intraventricular lesions. Neurosurgical Focus (1999) 6(4):e5. doi: 10.3171/foc.1999.6.4.8 16681359

[16] Qiao L Souweidane MM. Purely endoscopic removal of intraventricular brain tumors: a consensus opinion and update. Minimally Invasive Neurosurg (2011) 54(4):149–54. doi: 10.1055/s-0031-1284386 21922442

[17] Eliyas JK Glynn R Kulwin CG Rovin R Young R Alzate J. Minimally invasive transsulcal resection of intraventricular and periventricular lesions through a tubular retractor system: multicentric experience and results. World Neurosurg (2016) 90:556–64. doi: 10.1016/j.wneu.2015.12.100 26805678

[18] Najjar MW Azzam NI Baghdadi TS Turkmani AH Skaf G. Endoscopy in the management of intra-ventricular lesions: preliminary experience in the middle East. Clin Neurol Neurosurg (2010) 112(1):17–22. doi: 10.1016/j.clineuro.2009.08.027 19783360

[19] Cikla U Swanson KI Tumturk A Keser N Uluc K Cohen-Gadol A. Microsurgical resection of tumors of the lateral and third ventricles: operative corridors for difficult-to-reach lesions. J Neuro-oncology (2016) 130(2):331–40. doi: 10.1007/s11060-016-2126-9 PMC5090015 27235145

[20] Dănăilă L. Primary tumors of the lateral ventricles of the brain. Chirurgia (Bucharest Romania 1990) (2013) 108(5):616–30. 24157104

[21] Nakajo K Uda T Goto T Morisako H Nishijima S Kawashima T. Changes in cognitive function after resection of lesions in the anterior part of the lateral ventricle via an interhemispheric transcallosal approach. J Clin Neurosci (2020) 79:39–44. doi: 10.1016/j.jocn.2020.07.026 33070915

[22] He J Li Z Yu Y Lu Z Li Z Gong J. Cognitive function assessment and comparison on lateral ventricular tumors resection by the frontal transcortical approach and anterior transcallosal approach respectively in children. Neurosurgical Rev (2020) 43(2):619–32. doi: 10.1007/s10143-019-01088-2 30815764

[23] Deopujari CE Karmarkar VS Shaikh ST Mohanty CB Sharma V Tadghare J. Neuroendoscopy in the surgical management of lateral and third ventricular tumors: looking beyond microneurosurgery. Neurol India (2021) 69(6):1571–8. doi: 10.4103/0028-3886.333458 34979645

